# Exploration of an Integrated Active Learning Strategy to Balance Student Workload in a Mixed Level Research Methods Course

**DOI:** 10.15694/mep.2020.000269.1

**Published:** 2020-12-01

**Authors:** Stanley K Ellis, Taren M Swindle

**Affiliations:** 1 University of Arkansas for Medical Sciences

**Keywords:** Team Based Learning (TBL), Process Oriented Guided Inquiry Learning (POGIL), Transparent Expectations, Early Communication of Expectations, Team Role Assignments, Diligent Isolate, Peer Review

## Abstract

This article was migrated. The article was marked as recommended.

Team Base Learning (TBL) and Process Oriented Guided Inquiry Learning (POGIL) are two very distinct active learning and teaching pedagogies, both of which focus on team interactions to facilitate learning. TBL and POGIL literature have both been remiss in addressing their applicability in solving workload imbalance within the team paradigms respective of their individual pedagogy. In this study, we merged integral components of both TBL and POGIL teaching strategies to address perceived imbalance in student workload that were revealed through analysis of initial course evaluations from a mixed level (masters and doctoral level students) Research Methods course. As a result of findings from analyses of initial course evaluations, teams were established based on the integration of TBL and POGIL components in the subsequent course offering.

## Introduction

Concerns about America’s competitive standing among its international counterparts in training and retention of students for science, technology, engineering and math (STEM) disciplines has called for less traditional methods of teaching that will be effective within larger student groups ( Wood, 2009). Support for this argument has been based in educational research that has uncovered a need for new methods of knowledge transmission to large student groups where more traditional avenues have failed ( Hake, 1998; Wood, 2009). As a result of these two catalysts, several innovative teaching practices that are more student-centered, hands-on and active in their approach have been implemented and proven effective in increasing student learning outcomes ( Wood, 2009).

Learning occurs when students actively process the information and less so when they passively receive facts ( Lujan and DiCarlo, 2006). Knowledge retention is increased through active learning or by students actively engaging in the curriculum and taking on greater responsibility for their learning by accepting primary roles in the process ( Camiel *et al.*, 2016). Learning can be achieved with greater results by increasing the amount of collaborative learning activities available to students, opportunities for inquiry, critical thinking, and scholarly works ( Lujan and DiCarlo, 2006). Two approaches to active student learning include Process-Oriented Guided Inquiry Learning and Team Based Learning.

**Process-Oriented Guided-Inquiry Learning.** Process Oriented Guided Inquiry Learning (POGIL) is a student-centered teaching and learning pedagogy that is usually employed in science education ( Eberlein *et al.*, 2008). POGIL requires students to operate within self-managed teams during in-class time. With their teams, students navigate the three phases of the “guided inquiry” learning cycle: (a) exploration; (b) concept formation; and (c) application ( Eberlein *et al.*, 2008; Brown, 2010). During exploration, students examine a model (i.e. graphs, data, text, charts, illustrations, etc.), identify patterns, and glean knowledge from the model through the use of carefully developed questions. At the concept formation stage, students develop a concept based on their inquiry and findings from the model they encountered during the previous phase. In the application stage, students apply their new understanding of concepts to problems and their grasp of the concepts is thereby enhanced ( Eberlein *et al.*, 2008; Brown, 2010).

POGIL was borne in the chemistry discipline and was originally designed as a strategy that would replace traditional lectures ( Minderhout and Loertscher, 2007; Eberlein *et al.*, 2008; Brown, 2010). Through its implementation, the instructor facilitates the guided inquiry to help students arrive at the correct conclusions. By guiding their inquiry, the instructor also facilitates students’ problem solving, critical thinking, communication, human resources management, time management, and self-assessment abilities ( Eberlein *et al.*, 2008; Brown, 2010).

Modern researchers of learning recognize that student learning is optimized when students are engaged in meaningful social settings while being challenged to construct understanding of and solve complex problems ( Minderhout and Loertscher, 2007). The guided inquiry process, combined with teamwork and mastery of discipline specific content facilitate this process of student learning ( Minderhout and Loertscher, 2007). Classrooms that employ the POGIL pedagogy foster an environment where students can actively engage during in-class time. Better understanding of concepts, improved critical thinking, and increased ability to solve complex problems are facilitated by students’ active engagement in the learning process ( Moon *et al.*, 2016). POGIL takes into account students’ prior knowledge, including their misconceptions, and allows them to explore models, data, or illustrations through guided inquiry ( Brown, 2010). Students also realize the benefit of developing their teamwork skills by working together to solve complex problems while they fulfill the duties of their assigned team roles ( Moon *et al.*, 2016; Roller and Zori, 2017).

To facilitate team management, an instructor adopting POGIL assigns each team member a specific role. Although variations exist among proponents of POGIL in the team roles, the more commonly assigned roles are manager, recorder, reflector, technician, leader and/or presenter ( Eberlein *et al.*, 2008; Roller and Zori, 2017). Students’ performance of their assigned roles, in concert with the facilitation of the POGIL event, promotes the development of process skills beyond what is expected by the planned activities ( Eberlein *et al.*, 2008).

**Team Based Learning (TBL).** TBL is an active learning strategy that utilizes teams that are formed through a transparent and random process. TBL teams are made up of four to six members that remain together for the duration of a course. Teams follow a four-phase process: Pre-work/reading assignments; Individual Readiness Assurance Test (IRAT); Group Readiness Assurance Test (GRAT); and application exercise. In phase one (preparation), students are assigned pre-reading materials to review prior to class and are held accountable for the information through discussions with their teammates, the instructor and the Readiness Assurance Test (RATs). The RAT comprises phases two and three of TBL.

During phase two, each student takes a brief quiz, or IRAT, over the pre-reading material, without assistance from their teammates. In phase three, directly following the IRAT, each team engages in discussion to answer GRAT questions, which are identical to the IRAT. Mini-lectures are facilitated by the instructor to bring clarity to unclear points that may have remained from the RATs.

The collaborative learning that transpired during phases two and three are then applied by each team to solve real world problems in the application exercise phase ( Michaelsen, Knight and Fink, 2004; Michaelsen *et al.*, 2008). Throughout TBL, discussions and debates occur within groups and between groups with the instructor facilitating and directing the flow. This process is similar to that of POGIL. However, one important difference between TBL and POGIL is that student teams are not permanent for the duration of the course in POGIL pedagogy. Integration of team role assignments from POGIL into TBL learning has not been studied.

**Study Rationale.** An analysis of the 2015-2016 data from student end-of-course evaluations in a mixed-level (Ph.D. and Masters) graduate student survey research methods course revealed that both levels of students perceived an imbalance in the course workload assigned to their respective learner classification. Further analysis of student response data uncovered needed improvements in clarification of student course expectations and instructor responsibilities to the students. Both Ph.D. and Master’s level students described dissatisfaction with the volume of work performed by the other. To assuage students’ perceived inequities in workload among Ph.D. and Masters level students in the course, the investigators integrated relevant aspects of the process oriented guided inquiry learning (POGIL) and team based learning (TBL) teaching pedagogies. The intent was to integrate the two pedagogies and employ the new model in the research methods course as a strategy to transparently and adequately distribute work among both groups and increase course structure in the process. The study aimed to examine how integrating components from TBL and POGIL instructional pedagogies would impact (1) perception of imbalance in workload among Ph.D. and Master’s level students; and (2) students’ overall satisfaction with the course, two elements considered to support the overall learning experience. We hypothesized that the TBL and POGIL-driven changes would reduce course complaints and improve student course ratings.

## Methods

### Procedure

In fall 2015 (Year 1), a graduate-level Survey Research methods course was taught using a traditional lecture format. In fall 2016 (Year 2), the course was modified to include instructional components from POGIL and TBL teaching pedagogies within the context of project-based approach in which students applied learning topics across the semester to the design and execution of their own survey research project. A parallel mixed methods design was employed to evaluate students’ experiences in each approach. The collection of quantitative evaluation ratings and qualitative comments from students supports triangulation of our findings ( Creswell and Plano Clark, 2007). All evaluation data were collected at the end of the 16-week course. Ethical approval for this study was obtained from the [Blinded for Review] Institutional Review Board.

Course Redesign. Three major changes were implemented in our course redesign and consisted of: 1. Transparency and Early Communication of Expectations: During the first class of the newly redesigned Survey Research Methods course, the instructor dedicated an entire class period to verbally communicate to the students what they were expected to do to successfully complete the course. The expectations that were addressed included professionalism, contributions to team assignments, class attendance, work submissions, and active participation in the learning process. Students contributed to the articulation of the course expectations and were issued a copy in writing for their reference. The instructor also invited students to contribute to a set of expectations for their instructor. The instructor facilitated discussion around the expectations to ensure understanding by each student (for student and instructor expectations list see Appendix A). The syllabus was also redesigned to highlight dual course numbers. A five-hundred number indicated graduate students (master’s level) and a six-hundred number designated Ph.D. students. The instructor built discussion around the implications of both course numbers when reviewing the syllabus to ensure that both graduate and Ph.D. students understood the differences in workload, with additional course rigor attributed to Ph.D. students.

2. Team Role Assignments: In the first class period, students were assigned to permanent teams (TBL) which divided students based on their educational level (masters or doctoral) and field of study (e.g., health professions, biostatistics, health policy). Each team was composed of four to five team members (N = 5 groups); doctoral students were assigned a role and remaining team members decided their team role from a defined list based on strengths and interests. The team roles were defined for students in writing (POGIL). Role titles and duties were based on those similar to an actual research team which was in line with the overall concept of the course. The instructor clarified each role and its associated responsibilities to improve students’ understanding of their assigned tasks and role requirements. Team roles included: (A) Principal Investigator; (B) Research Associate 1 (Data Collection Focus); (C) Research Associate 2 (Presentation Focus); (D) Research Analyst; and (E) Project Consultant (roles and descriptions found in Appendix B). A Ph.D. student was assigned to each team. As the most senior students on each team, based on their educational level, Ph.D. students were assigned the Principal Investigator’s role for each team to give them leadership experience they could take with them into the workforce. To facilitate transparency of team assignments, all team roles were established during the first day of class. Team roles (excluding PI role) were negotiated among the team members. Adjustments were made as necessary by the instructor to facilitate teams’ composition.

3. Peer Review: In accordance with TBL pedagogy, the redesigned course included a greater emphasis on structured peer review ( Michaelsen *et al.*, 2008). Both courses included a mid-semester PowerPoint presentation of their research plan and an end- of-the-year poster presentation of their findings. Three primary changes were made to improve this process. First, students in the revised course were required to submit their presentations early to the online learning system for peer review. Second, students were asked to come to the presentations prepared with clarifying questions for their classmates. Finally, the quality of the peer review (completed with the instructor’s rubric) was the basis for a participation grade for the day.

### Participants

All participants in this study were graduate students in a College of Public Health. In fall 2015, 10 were Masters level students and 7 were doctoral students. In fall 2016, 17 were Masters level students and 5 were doctoral students. Students were enrolled in graduate programs in epidemiology, health behavior and health education, health administration, healthy policy and management, and nursing. To ensure anonymity in responses no demographic information was collected for students.

### Measures

*Departmental Course Evaluation.* Each year the College of Public Health distributes student evaluations through the online learning platform for all courses in the department. Students are encouraged to complete the evaluations through emails from department administration, not by the course professor. Students receive email reminders until the evaluations are complete. The departmental evaluation includes both an instructor evaluation and a course evaluation. The instructor evaluation includes an overall rating and ratings in 7 topic areas: organization, clarity, enthusiasm, contributions, rapport, professionalism, and attitude. The course evaluation includes and overall rating and ratings questions in 5 topics: organization, clarity, content, materials, and fairness. All quantitative items were rated on an 5-point scale (1 = Strongly Disagree, 5 = Strongly Agree). Each of these evaluations invited open-ended comments and suggestions from students. In total, 82% of Departmental Course Evaluations were completed in fall 2015; 76% were completed in fall 2016.

*Professor Course Evaluation.* The professor of the course [initials blinded for review] developed a course evaluation more specific to the expectations and objectives of the course for 2016. Students rated the degree to which collaboratively developed course expectations and learning objectives were achieved on a 1 to 4 scale. (1 = Strongly Disagree, 4 = Strongly Agree). Items focused on expectations of peers (e.g., “We listened to one another, N = 9) as well as expectations of the course instructor (e.g., She was consistent, N = 15). Open-ended questions (N = 5) asked students to describe (1) distribution of work among the team, (2) differences and similarities to other experiences with being on a team in courses, (3) contributions of the team science project to their learning, (4) suggestions for improving their learning on the team science project, and (5) suggestions for the instructor to improve the team science project. All students in the class completed this evaluation (i.e., 100% completion).

### Data Analysis

Quantitative

Data were imported into SPSS (IBM) for analysis. Means for items on all quantitative questions were computed for each school year for the professor-developed measures and were provided for the departmental evaluation. Scales were created by averaging items in the same topic area. Comparisons between items and scales were made using independent-sample t-tests.

Qualitative

All student comments were imported into Nvivo (2012, Version 10) in Microsoft Word (.doc) format for further analysis according to the textual data preparation guidelines for Nvivo. A single coder [initials blinded for review] completed the qualitative coding process. Initial analysis of student survey response data resulted in 11 categories. The final coding results became the coding scheme for the data. The 11 categories were winnowed into meaningful chunks of data, resulting in two categories and three sub-categories that became the coding scheme for the student data.

## Results/Analysis

Quantitative

*Departmental Course Evaluation.*

**Figure 1.**
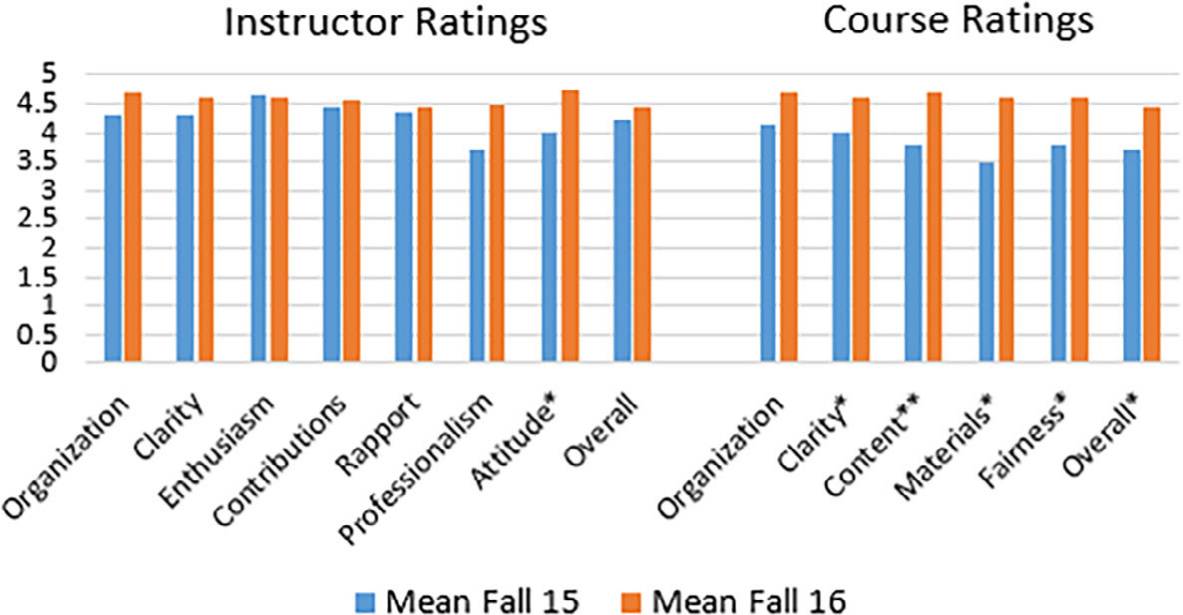
Mean comparisons and T-test results for Departmental Evaluations. * *p* <.05, ** *p* <.01

Instructor. Only one of 7 ratings of the instructor was significantly different between semesters; attitude, *t*(28) = 2.37, *p* = .03. However, all ratings either stayed the same or increased between the original and revised semester. See Figure 1 for means and t-test comparison results.

Course. Significant differences ( *p* ≤ .05) were observed between ratings for 4 of 5 topic areas and for the overall rating when comparing the lecture-based and POGIL/TBL-based courses. In every case, means were higher for the revised semester reflecting POGIL and TBL changes. Mean increases on the 5-point scale ranged from 0.54 for instructor organization (not significant) to 1.13 for instructor materials (p = .04). See Figure 1 for means with significance of t-test comparisons indicated.

*Professor Course Evaluation.* For Year 2,all items evaluating peer expectations averaged 3.2 (out of 4) or greater. Still the lowest rated item ( *M* = 3.2) was, “My peers avoid controlling the group activities.” The highest rated item ( *M* = 3.6) was, “Were respectful of others and their ideas.” For items related to the expectations of the instructor all were at or above an average of 3.3 or greater (out of 4), which was observed for the item “The instructor avoided assignments that were just busy work.” The highest means for the instructor expectation ( *M* = 3.9) was “The instructor shared knowledge.”

Qualitative

**Year 1.** Year 1 produced a primary code of “student grievances” described as students’ expressed discontent with the course instructor, class operations and management or teammates’ behaviors/performance. Exemplars of student grievances included academic rigor and workload distribution, which emerged as the two dominant sub-codes resulting in seven and six occurrences in the data, respectively ( Table 1). Based on the thematic description provided by students’ responses, academic rigor was coded as the amount, frequency and complexity of tasks. Students also described concerns with the distribution of work tasks among Ph.D. and Masters students within teams. This descriptor provided the basis for the workload distribution category.

**Table 1.** Year 1 Primary Code: Student Grievances

Sub-codes Exemplar Quotes		
	Academic Rigor	Workload Distribution
	The amount and frequency of quizzes and assignments probably led me to truly comprehend less of the content, and just focus on the quiz questions or specific assignment.	Weekly readings with discussion posts and sporadic assignments on top of the group project assignments was a lot of weekly work for this course.
	Too much work for one course. Reading responses were every week, in addition to that, there were assignments sometimes. In addition to the text book, there were more than 1 articles assigned to the each reading.	Even though it is expected that they would have more skillsets to effectively lead, more emphasis should have been placed on including MPH students even more.

During the first year iteration of the course, students expressed discontent with what they perceived as unrealistic course expectations and over rigorous assignment and grading practices. Despite student grievances, instructor accolades appeared as the second most common theme from the analysis. Year 1 accolades included comments regarding faculty responsiveness, grading practices, feedback, availability and fairness ( Table 2).

**Table 2.** Secondary Code for Year 1 and 2: Instructor Accolades

Exemplar Quotes	Year 1	Year 2
	I think as a teacher, you did an excellent job (responded quickly to emails/grading, gave additional information for topics outside of class, had good slides for taking notes, etc.)	Dr. [name removed] is great. Don’t let her go anywhere. So energetic and ready to share her knowledge. She is very good with students.
	Explained the material very nicely and answered all the questions. Always gave prompt feedbacks.	Dr. [name removed] is the best. She kept us all engaged during classes. Students might say she is strict, but I think she is fair and a person with principles. She is very knowledgeable, humble and down to earth. She takes her job as an instructor very seriously, and she cares about her students. She treated all of us as family. I would take her class a million times :)

**Year 2.** Analysis of year two data resulted in a thematic shift to instructor accolades ( Table 4) as the primary occurrence and student grievances ( Table 3) as secondary. In the second year iteration of the course, workload distribution did not emerge as a dominant sub-code. Workload distribution was expressed in terms of academic rigor, which was identified in an equal number of references as classroom management and described the instructor’s management of activities, behaviors, and classroom processes. Examples of academic rigor included: Instructor accolades, which were described as praises for the teacher’s performance and instruction of the course. The main focus of students’ grievances was on the sub-code of grading practices. Students described the instructor’s grading practices as capricious and unnecessarily strict ( Table 3).

**Table 3.** Year 2 Secondary Code: Student Grievances

Sub-code Exemplar Quotes	Grading
	Her assessment of the professionalism grade appears arbitrary as no feedback is offered. I don’t think the professionalism grade is necessary; it’s a bit demeaning.
	However, the fact that everything was graded (very seriously) made the course quite stressful.
	Dr. [name removed] does not seem to respect the achievements that her students possess coming into the course. This is a graduate level course with a combination of PhD and master level students. Further, there are students who have medical degrees, masters degrees, and many working professionals. Yet she feels it necessary to grade on “professionalism.”

Year two data materialized an almost even distribution of references towards social loafing and workload distribution, respectively. Unexpectedly, the phenomenon of diligent isolates was observed in the data ( Table 4). Diligent isolates were characterized as team leaders who take over in a group and work independently, while deliberately or inadvertently discourage contributions from their team members ( Pieterse and Thompson, 2010). References to social loafing could be skewed as a result of the diligent isolate phenomenon ( Lam, 2015).

**Table 4.** Diligent Isolate Phenomenon

**Exemplar Quotes**	Most of the work fell on me. I did nearly all of the writing and I had to practically demand research/input from girls, which I never got until the day assignments were due.
	I enjoyed my experience, but I would honestly prefer to have a group with only Mph students. While [team member 1] and [team member 2] taught us a lot, they have more expertise and left [team member 3] and I out.
	I think our PHD students should have been better at being open to others schedules, and distributing tasks was a problem because the PHD students would complete the work w/o other member’s feedback.

## Discussion

This study investigated the perceptions of workload imbalance held by both masters and Ph.D. students in a mixed level, graduate research methods course. Components from both TBL ( Michaelsen *et al.*, 2008) and POGIL ( Roller and Zori, 2017) active learning pedagogies were introduced into the curriculum to mitigate the perceptions of workload imbalance among the two learner groups. Three salient themes emerged during the study: (1) diligent isolates; (2) student efficacy with the new curriculum; and (3) increased student satisfaction due to enhanced clarity of expectations and improved distribution of work assignments among team members.

Analysis of students’ narrative survey responses for both Years 1 and 2 discovered two divergent themes. Academic Year 1 course data revealed a perception of social loafing attributed to the masters level students, primarily by their Ph.D. counterparts. However, academic Year 2 course data uncovered a common thread in the masters students’ description of Ph.D. students’ in-group and leadership behaviors. Ph.D. students were characterized by their masters level team members as “lone wolves” ( Barr, Dixon and Gassenheimer, 2005), indicative of taking control of and completing assignments alone and without accepting or soliciting feedback or input from their teams. The diligent isolate behaviors were described by the masters level students as a hindrance to team cohesion, productivity, and overall morale among the teams.

A review of the course ratings for Year 1 and Year 2 revealed significant changes in the overall course ratings. However, the instructor ratings remained essentially the same. Although our study did not include a control group, this suggests that improvements in course ratings were not confounded with instructor ratings. That is, it does not seem probable that the course rating differences were attributable to different student perceptions of the instructor. Course ratings have been questioned as an important predictor of student learning ( Uttl, White and Gonzalez, 2017); however, course ratings have also been associated with positive student outcomes such as autonomy, competence, and feelings of relatedness to the course rated ( Filak and Sheldon, 2003). Further, courses heavy in quantitative components (like research methods) have been associated with lower student evaluations and thus lower career achievement for professors who taught those courses ( Uttl and Smibert, 2017). Thus, it behooves both students and professors to identify classroom strategies to improve students’ experiences and engagement in courses perceived to be difficult. In our study, improvements across the two years and consistently strong evaluations in the second year (all ratings > 3 out of 4) suggest that providing clear structures to course projects may be important to this end.

Our findings suggest a primary reason for improved student satisfaction with the course was improved clarity in expectations and greater transparency with the duties assigned in distribution of the workload. This is consistent with previous studies which describe how instructors’ failure to clearly communicate student expectations compounds the effects of social loafing in groups and leads to greater student dissatisfaction ( Hall and Buzwell, 2013; Lam, 2015). A review and analysis of prior year survey data and qualitative comments for the course revealed student grievances with and lack of understanding surrounding what was expected of them. As a result, transparency and early communication of course expectations was addressed with greater attention to detail during the first class. This is a strategy supported by POGIL pedagogy ( Eberlein *et al.*, 2008; Soltis *et al.*, 2015). To our hypothesis, adoption of team role assignments for each team member seemed to increase the clarity and transparency of individual and group expectations and contribute to students’ overall satisfaction. In accordance with our POGIL-driven strategy ( Brown, 2010; Roller and Zori, 2017), grievances regarding miscommunications of expectations and lack of understanding with course requirements were reduced significantly from the previous years’ courses survey responses.

## Conclusion

The qualitative and quantitative results of this study converge to support improved students’ satisfaction with the distribution of work assignments between masters and Ph.D. students during the second iteration of the course, over prior year. Although these two pedagogical teaching approaches are very similar in their processes, very distinct components of each were integrated as a strategy to enhance course structure and reduce the perceptions of workload imbalance among Ph.D. and Masters level learners in our research methods course. The new teaching and learning paradigm proposes that students be involved in their learning to internalize it ( Bluestone, 2007; Schultz, Wilson and Hess, 2010). Several strategies to achieve this within a classroom include deliberate seat arrangement, group breakout sessions, class discussions, etc. ( Pepper and Pathak, 2008). Assigning students roles within their assigned teams and requiring them to exact the written duties of those roles based on the content and objectives of the course would fit within the new educational paradigm of active classroom learning, especially the POGIL paradigm ( Eberlein *et al.*, 2008). Students’ descriptions of unrealistic faculty expectations of their course performance and overly complicated assignments disappeared in the second course iteration. Contributions to the student efficacy could be attributed to the improved efforts by the course faculty to communicate course expectations and assign each student to a specific and “real world” team role that was attached to equally specific team member duties.

Although this study revealed important themes that can be acted on to improve future iterations of the course, there were several limitations to the study. Conclusions described in this study are based on only one iteration of the research methods course where the intervention was applied in a small sample of students. There is a lack of historical data and literature that addresses comparison of these two groups of learners in a similar setting. Although the study was a mixed methods approach, randomization of participants to their respective groups did not take place. This study did not address any relationships between the early communications of course expectations and student outcomes in comparison to prior year. Future research can examine the unique and interactive effect of two key changes we incorporated into our study but were unable to study apart from other changes: (1) early, explicit communication of course expectations and (2) diligent isolate phenomena within student teams.

## Take Home Messages

• Unclear or lack of course/instructor expectations can impede faculty teaching and student learning.
• Assigning students specific team roles with clearly outlined duties may contribute to student satisfaction with their learning experiences.
• Students express fewer complaints with their learning experience when faculty clearly communicate expectations and assign student to specific roles with “real world” applications.

## Notes On Contributors

**Dr. Stanley K. Ellis** is an Assistant Professor at the University of Arkansas for Medical Sciences and currently serves as Director of Education in the College of Medicine’s Institute for Digital Health and Innovation. Dr. Ellis was instrumental in helping to create a faculty and resident development series on education for the Department of OB/Gyn. ORCID ID: https://orcid.org/0000-0002-1431-0494.

**Dr. Taren M. Swindle** is an Assistant Professor in Family and Preventive Medicine within the College of Medicine at the University of Arkansas for Medical Sciences. She received her Ph.D. in Educational Psychology and Research from the University of Memphis. Her research program focuses on measurement development and increasing adoption of evidence-based practices and interventions in community settings such as this through application of Implementation Science. ORCID ID: https://orcid.org/0000-0001-7231-6002.

## Appendices

### Appendix A

Survey Research Methods Course Expectations List

Expectations of Students

• Timely and effective participation.
• Be present and contribute to class discussions.
• Be respectful of others’ opinions/perspectives.
• Participate in learning. Be an active learner.
• Turn work in on time.
• Support others’ work.
• Contribute equally on student projects.
• Give thoughtful responses to discussion questions.
• Share relevant background/experiences.

Expectations for Professor

• Respect students’ opinions
• Provide timelines and be prepared.
• Be organized.
• Be aware of different levels of knowledge about this material.
• Provide meaningful assignments.
• Come on time.
• Return grades on time.
• Communicated clearly and effectively about assignments, grades, and projects.
• Challenge students.
• Make class as practical and relevant as possible.
• Teach how and when to use surveys.
• Allow students to contribute in their own way as long as they meet course requirements.
• Provide practical and relevant examples of data that relate to the materials we cover.
• Provide opportunities to review before we have a large project.

### Appendix B

Survey Research Course: Individual Team Member Roles

**The Principal Investigator** (PI) provides general oversight of project activities working to ensure positive team climate, quality of work product, and compliance with project deadlines. Additionally, the Principal Investigator is responsible for ensuring integrity of the design, conduct, and reporting of the research project and compliance with course policies and expectations. The PI will coordinate the development of the research protocol and instrumentation with input from team members. Further, the PI will lead the team in deciding how other roles will be assigned and provide the instructor with a list of role assignments for the group by Week 2.

**Research Associate** 1 (Data Collection Focus) conducts independent and collaborative research tasks including, but not limited to, coordination of creation of online survey forms, recruitment, data screening, and cleaning. RA 1 will work closely with the Research Analyst to ensure data quality as well as to construct a survey codebook consistent with course expectations. RA 1 will coordinate and incorporate team feedback into assigned tasks and contribute to all writing assignments.

**Research Associate** 2 (Presentation Focus) conducts independent and collaborative research tasks including, but not limited to, coordination of creation of presentation outlines/notes as well as Power Point slides for oral and poster presentation. RA 2 will lead the development of a plan for survey testing during the course presentation. RA 2 will coordinate and incorporate team feedback into assigned tasks and contribute to all writing assignments.

**The Research Analyst** manage and analyze data; research, collect and analyze data, solve problems and communicate findings in summary reports. The analyst will ensure accurate and compliant collection, storage, and entry of data. Further, the analyst will lead efforts to manipulate and analyze data (create scales, reliability, descriptive statistics).

**The Project Consultant** provides content expertise in the survey topic area. Thus, the team should agree to conduct their survey in a research topic area about which the project consultant has some existing knowledge and interest. The project consultant will lead the team in identifying seminal and recent articles related to the chosen topic as well as identifying potential existing measures to inform the survey design. The consultant will contribute to other team tasks and writing assignments.

## Declarations

The author has declared that there are no conflicts of interest.

## Ethics Statement

The University of Arkansas for Medical Sciences did not deem this Human Subjects Research. Ethical approval for this study was obtained from the [Blinded for Review] Institutional Review Board, Reference #206683.

## External Funding

This article has not had any External Funding
