# Supplementary material for: Exploration of an Integrated Active Learning Strategy to Balance Student Workload in a Mixed Level Research Methods Course
Source: MedEdPublish (2016). 2020 Dec 1;9:269. [Version 1] doi: 10.15694/mep.2020.000269.1 (PMC10697459; doi:10.15694/mep.2020.000269.1)
Supplement: Peer review history

# Reviewer report: Reviewer response for version 1

**DOI:** 10.21956/mep.20139.r31099

**Published:** 2021-01-06

**Authors:** Megan Anakin

**Affiliations:** 1 University of Otago

This review has been migrated. The reviewer awarded 2 stars out of 5 Thank you for writing this research article about examining how learning can be enhanced for postgraduate research students in health professional education.While the title and abstract uses language that suggests a constructivist framework for the study, the first paragraph contains terminology that suggests a positivist approach, specifically, the phrase, “uncovered a need for new methods of knowledge transmission”. Please describe the traditional methods of teaching that are problematic and how they have failed so the warrant for using innovative teaching practices that have been proven to be effective in student learning is well established. Please provide examples of how collaborative learning has resulted in greater learning from the literature that may be applicable to your teaching area. To enhance the explanations of the POGIL and TBL pedagogical approaches, please give an example in the context of the study to help the reader appreciate the potential applicability of those methods to your learners and subject matter. Please provide references for the explanation of TBL and please consider revising the explanation of the four phase process because there are several abbreviations used and the phases are not described in a linear order.In the paragraph about the study’s rationale, data from 2015-2016 are discussed. Please clarify if these data are the same or not as the data analysed in the study. It appears that the findings of the first year of the study have been used to justify the warrant of the study. Please consider if it is appropriate to justify the study with findings and/or data that have not yet been reported in the same article you are writing. Pleases consider including a statement that provides evidence that POGIL and TBL may be appropriate to address the concerns raised in the evaluation data.Please remove replace the phrases ‘Blinded for Review’ with the missing information.In the methods section, please consider revising the paragraph about the team role assignments to remove the repeated information. Please move the numbers of participants and response rates to the results section. Please note that demographic information reported in aggregate (gender, ethnicity, age or work experience) can be collected with ensuring anonymity without revealing a participant’s identity. In the quantitative data analysis paragraph, please state the construct that was used to provide the rationale to justify combining items to form a scale. Please report the means and standard deviations for each item and scale in a Table instead of the Figure used to show complete results. In the qualitative data analysis paragraph, please describe and reference the data analysis methods used that results in the frequency of codes being reported as results. Please either give the participants pseudonyms or random alphanumerical identity codes (such as P1Doctoral, P2Masters) so the reader can see that the quotations do not all come one participant. In the discussion section, please include a paragraph about the methodological limitations of this study.I would be very happy to read and review a revised version of this article.

Reviewer Expertise:

NA

No decision status is available

# Reviewer report: Reviewer response for version 1

**DOI:** 10.21956/mep.20139.r31100

**Published:** 2020-12-21

**Authors:** Anita Samuel

This review has been migrated. The reviewer awarded 3 stars out of 5 Group/collaborative work is always challenging, and the common grievance is workload distribution. You provide an interesting approach to addressing this issue. Thank you for providing your resources in the appendices. That is very helpful for others who might want to replicate this approach. Currently, the abstract seems incomplete. Your abstract mentions the intervention but does not include any information about the findings from this study. A couple of sentences about this would provide a more comprehensive description of the paper.The reporting of the qualitative data does not align with the key focus of this paper – workload distribution between team members. In Table 1, the quotations under Workload Distribution address overall course workload distribution rather than the team dynamics. None of the data presented from Year 1 substantiate the rationale for the study. Interestingly, the data in Table 4 for Year 2 suggest the team workload imbalance. Since you provide instructor ratings, it would be helpful to clearly state if the same instructor taught both Year 1 and Year 2 offerings of the course. Given the feedback you have received from Year 2 participants, are you planning any changes to the next iteration of this course?The Professor Course Evaluation includes a question about suggestions for the instructor to improve the team project. It would be helpful to know how participants responded to this question since improving the team project experience is the focus of this paper. Combining POGIL and TBL is an innovative approach which has application implications beyond medical and health education. This would be a stronger paper if the above-mentioned issues were clarified.

Reviewer Expertise:

NA

No decision status is available

# Reviewer report: Reviewer response for version 1

**DOI:** 10.21956/mep.20139.r31101

**Published:** 2020-12-09

**Authors:** Samar Aboulsoud

**Affiliations:** 1 CAIRO UNIVERSITY, SCHOOL OF MEDICINE

This review has been migrated. The reviewer awarded 4 stars out of 5 Dear Authors,Thank you for sharing this very interesting article. I enjoyed reading it and learning about your innovative, hybrid approach. I have few queries and comments that might be useful for further adoption of your model.1. Did you face any challenges with the introduction of a new curricular model in the middle (year 2) of the course? Do you think that this would have worked better if the newly introduced model is applied to a new cohort of students? How did the students perceive /accept the change of the educational approach in the middle of their course? Did this approach yield better learning outcomes?2. What are the modifications suggested or introduced in your hybrid approach based on the students’ feedback e.g., diligent isolate phenomenon? 3. I am interested to learn more about the steps of the integration process i.e., integration of roles of team members in TBL within the phases of POGIL.Finally, I believe this approach might be an innovative solution that can help address many challenges that are faced with traditional curriculum implementation.Thank you again for sharing your experience.

Reviewer Expertise:

NA

No decision status is available
